# P-1697. Performance of the VITEK 2 with Advanced Expert System as a fast method for determination of antimicrobial susceptibility against 400 Enterobacterales isolates from North and Latin America (2022)

**DOI:** 10.1093/ofid/ofaf695.1870

**Published:** 2026-01-11

**Authors:** Michael D Huband, Paul Rhomberg, Allison Fuhrmeister, Christopher Blankers, Mariana Castanheira

**Affiliations:** Element, North Liberty, IA; Element Materials Technology/Jones Microbiology Institute, North Liberty, Iowa; Element Materials Technology/Jones Microbiology Institute, North Liberty, Iowa; Element Materials Technology/Jones Microbiology Institute, North Liberty, Iowa; Element, North Liberty, IA

## Abstract

**Background:**

The VITEK 2 with Advanced Expert System (AES) performs fast identifications and susceptibility testing. AES is based on an extensive, evolving database of MIC distributions/phenotypes, designed to analyze VITEK 2 results for biological validity, infer resistance mechanisms, and provide comments/corrections by labeling them as green (consistent), yellow (consistent with correction), and red (inconsistent). This study evaluated the performance of AES versus reference broth microdilution (BMD) against 400 Enterobacterales isolates from North and Latin America (2022).

**Figure:**
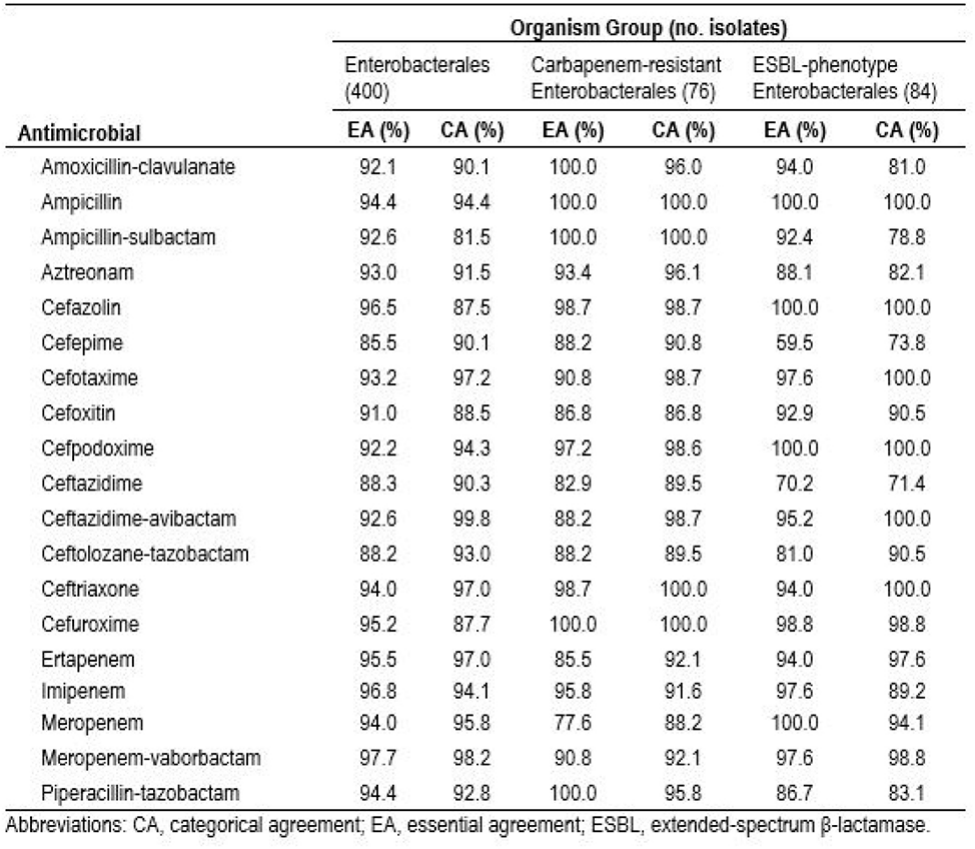
Organism Group

**Methods:**

Enterobacterales isolates included 56% wildtype, 19% containing carbapenemases, 21.2% containing ESBLs, and 3.8% with transferrable AmpC (tAmpC) genes. Isolates were tested by BMD following CLSI guidelines. BMD and VITEK 2 MIC results were compared for 30 antimicrobials. Discordant results were repeated by both methods. AES phenotypes were compared to resistant genotypes. AES green, yellow, and red categories were compared to reference BMD.

**Results:**

Essential agreement (EA) and categorical agreement (CA) rates for 19 β-lactam antimicrobials against the 400 Enterobacterales isolates were >90.0% for 16/19 and 15/19 of the tested agents, respectively, with AES rules enabled (Table). EA and CA rates were >90% for 12/19 and 15/19 of the β-lactam agents tested against carbapenemase containing strains, respectively and 14/19 and 12/19, respectively, for the ESBL containing strains. AES phenotypes were accurately reported for 98.7%, 97.6%, and 100.0% of the carbapenemase, ESBL, and tAmpC isolates. 311 isolates (77.8%) were reported by AES as green, 60 (15.0%) as yellow, and 29 (7.2%) as red. For AES green results, 99.0% (308/311) were correctly classified as wildtype, carbapenemase, ESBL, or tAmpC and could be automatically reported without further review. Among AES yellow results, corrections were acceptable for 53.3% (32/60). AES red results were not a match for 29/400 genotypes.

**Conclusion:**

AES green results displayed accurate phenotypes for 99.0% of the Enterobacterales isolates and could be confidently and quickly communicated (auto-posted) to clinicians, which could support both antimicrobial stewardship initiatives and better patient outcomes.

**Disclosures:**

Michael D. Huband, BS, Melinta Therapeutics: Advisor/Consultant|Melinta Therapeutics: Grant/Research Support Mariana Castanheira, PhD, Melinta Therapeutics: Advisor/Consultant|Melinta Therapeutics: Grant/Research Support

